# TFF1 activates p53 through down-regulation of miR-504 in gastric cancer

**DOI:** 10.18632/oncotarget.2156

**Published:** 2014-07-01

**Authors:** Mohammed Soutto, Zheng Chen, Mohamed A. Saleh, Ahmed Katsha, Shoumin Zhu, Alexander Zaika, Abbes Belkhiri, Wael El-Rifai

**Affiliations:** ^1^ Department of Veterans Affairs, Tennessee Valley Healthcare System, Nashville, Tennessee, USA; ^2^ Department of Surgery, Vanderbilt University Medical Center, Nashville, Tennessee, USA; ^3^ Clinical Pharmacology, Vanderbilt University Medical Center, Nashville, Tennessee, USA; ^4^ Department of Cancer Biology, Vanderbilt University Medical Center, Nashville, Tennessee, USA; ^5^ Department of Pharmacology and Toxicology, Faculty of Pharmacy, Mansoura University, Mansoura, Egypt

**Keywords:** TFF1, p53, gastric cancer, miR-504, apoptosis

## Abstract

The expression of TFF1 is frequently down-regulated in human gastric cancer whereas its knockout leads to the development of gastric adenomas and carcinomas in mouse models. The molecular mechanisms underlying the TFF1 tumor suppressor functions remain unclear. In this study, we demonstrate, using colony formation assay and Annexin V staining, that reconstitution of TFF1 expression in gastric cancer cell models suppresses cell growth and promotes cell death. Furthermore, using a tumor xenograft mouse model of gastric cancer, we demonstrated that reconstitution of TFF1 suppresses tumor growth *in vivo*. The results from PG13-luciferase reporter assay and Western blot analysis indicated that TFF1 promotes the expression and transcription activity of the p53 protein. Further analysis using cycloheximide-based protein assay and quantitative real-time PCR data suggested that TFF1 does not interfere with p53 mRNA levels or protein stability. Alternatively, we found that the reconstitution of TFF1 down-regulates miR-504, a negative regulator of p53. Western blot analysis data demonstrated that miR-504 abrogates TFF1-induced p53 protein expression and activity. In conclusion, the *in vitro and in vivo* data demonstrate, for the first time, a novel mechanism by which the tumor suppressor functions of TFF1 involve activation of p53 through down-regulation of miR-504.

## INTRODUCTION

TFF1 (formerly known as pS2) is a small-secreted protein that belongs to the family of trefoil peptides, and characterized by the presence of one to six cysteine-rich P domains [1]. In human primary gastric tumors, there is a frequent loss of TFF1 expression whereas disruption of the mouse *Tff1* gene leads to the development of gastric dysplasia and carcinomas in mice [2, 3]. In contrast, transgenic mice that overexpress *Tff1* have shown resistance to intestinal damage [4]. The function of TFF1 was first controversially considered as an oncogene in breast cancer [5, 6]. However, Buache and colleagues [7] demonstrated that TFF1 was not an oncogene in the mammary epithelium, but rather reduces the development of breast tumors and has a tumor suppressor function. Using a *Tff1* knockout mouse model, we and others have provided evidence supporting the tumor suppressor function of TFF1 and its anti-inflammatory role in gastric cancer [3, 8, 9]. While there are evidences showing that TFF1 suppresses growth and induces apoptosis in gastric cancer cells [10-12], one study has shown that recombinant TFF1 could have anti-proliferative and anti-apoptotic effects in gastrointestinal cells [13]. The mechanisms by which TFF1 might exert its tumor suppressor functions and promote cell death remain largely unrecognized.

The classical tumor suppressor gene, p53, plays a central role in maintaining genomic stability and preventing tumor formation [14-16]. Activated p53 selectively transcribes its target genes to start various cellular responses. The ability of p53 to induce cell cycle arrest, apoptosis or senescence is vital to prevent the propagation of damaged cells with harmful DNA mutations that could potentially become cancerous [16-18]. In more than 50% of human cancers, p53 is directly inactivated by mutations, and in the majority of the remaining cancer cases p53 activity is compromised by other mechanisms [15, 19]. Under normal conditions, p53 is a short-lived cell protein, which is maintained at a low level by rapid degradation through MDM2, a p53-specific E3 ubiquitin ligase [20, 21]. Under cellular stress conditions, p53 activation involves stabilization of the protein, and enhancement of its DNA binding and transcriptional activity. These changes in p53 are mediated by extensive post-transcriptional and post-translational modifications of p53 and protein-protein interactions with cooperating factors [22].

MicroRNAs (miRNAs) are non-protein-coding sequences considered as a novel class of potential biomarkers or therapeutic targets, and are thought to regulate the expression of more than 70% of human genes [23]. miRNAs regulate cell proliferation, apoptosis and metabolism, all of which are frequently altered in cancers [24]. Recent studies have reported that the regulation of the expression of specific miRNA could be an additional mechanism for p53 in tumor suppression. For instance, miR-34 was identified as a direct p53 target that regulates apoptosis, cell-cycle arrest, or senescence, contributing to tumor suppression [25-27]. In contrast, other microRNAs display oncogenic activities, promoting tumor progression. Hu and colleagues [28] identified a novel miRNA, miR-504, which acts as a negative regulator of p53 expression through its binding to two sites in p53 3′-UTR. Hence, overexpression of miR-504 inhibits the transcriptional activity of p53 and decreases the p53-induced apoptosis and cell-cycle arrest in response to genotoxic stress, suggesting that miR-504 may be implicated in tumorigenesis.

In this study, we identified a novel mechanism by which TFF1 suppresses cell growth and induces apoptosis through activation of p53, by down-regulation of miR-504, a negative regulator of p53, in gastric cancer cells.

## RESULTS

### Reconstitution of TFF1 expression inhibits growth in gastric cancer cells

**Figure 1:**
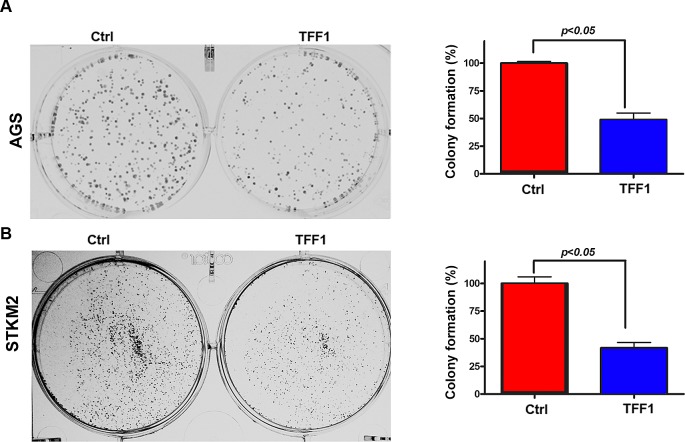
Reconstitution of TFF1 expression suppresses cell growth *in vitro* Representative images of colony formation assay in AGS (A) and STKM2 (B) cells stably expressing empty pcDNA vector (ctrl) or TFF1. Quantitative analysis of colony numbers is shown in the right panel. The data are expressed in percentage relative to control cells, and are representative of three different experiments.

TFF1 has been reported to be a tumor suppressor in gastric epithelial cells [1, 3, 9, 29, 30]. To determine whether TFF1 suppresses growth in gastric cancer cells, we generated two gastric cancer cell lines, AGS and STKM2, stably overexpressing TFF1 or control empty vector pcDNA, and performed colony formation assay. Our data indicated a significant reduction in colony formation in TFF1-expressing AGS (p<0.05, Figure 1A) and STKM2 (p<0.05, Figure 1B) cells as compared to control cells. These results clearly indicated that TFF1 stable expression significantly inhibits growth in gastric cancer cells.

### TFF1 activates p53 through down-regulation of miR-504

**Figure 2:**
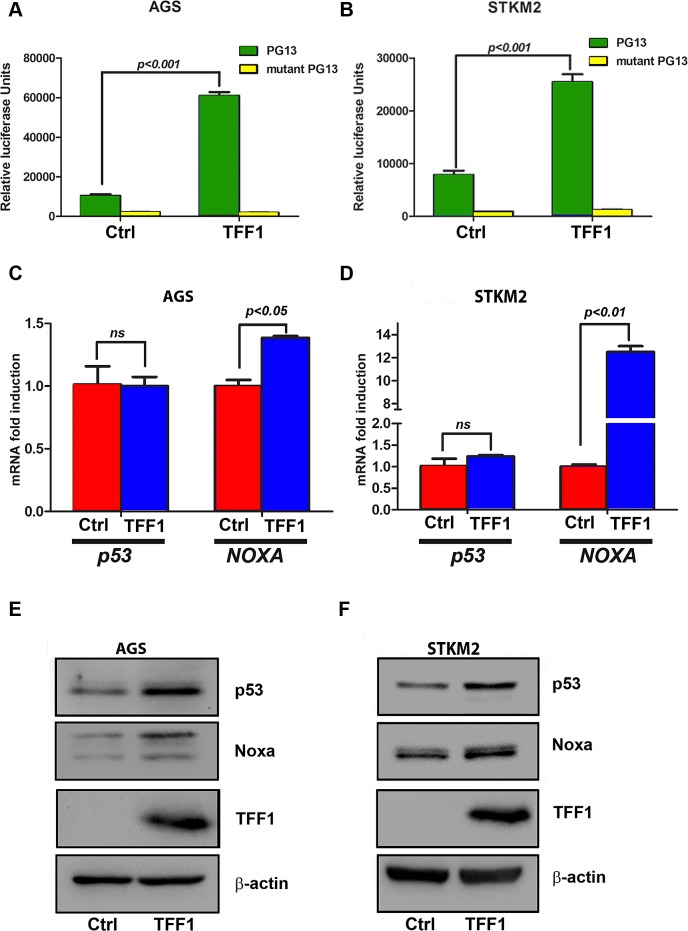
TFF1 increases p53 transcriptional activity and protein expression in gastric cancer cells The luciferase assay was performed on AGS (A) and STKM2 (B) cells that were transfected with TFF1 or PTT5 empty vector in combination with PG13-Luc or mutant PG13-Luc. The reconstitution of TFF1 expression increased the transcriptional activity of p53 in AGS and STKM2 cells. AGS and STKM2 cells were transiently transfected with TFF1 or PTT5 empty vector. Quantitative real time PCR and Western blot analysis were performed 48 h post transfection. The reconstitution of TFF1 expression increased *NOXA*, but not *p53*, mRNA levels in AGS (C) and STKM2 (D), and increased NOXA and p53 protein levels in AGS (E) and STKM2 (F) cells. These data are representative of three different experiments.

Suppression of cell growth and induction of cell death is usually associated with activation of p53 [31, 32]. To determine whether TFF1 regulates p53, we examined the effect of TFF1 on the transcriptional activity of p53 using the PG13-Luc reporter that contains multiple repeats of the p53 consensus-binding site. AGS and STKM2 gastric cancer cell lines were transiently transfected with PG13-Luc or mutant PG13-Luc along with TFF1 or PTT5 empty vector for 48 hours. Our results indicated a significant increase of p53 transcriptional activity in TFF1-expressing AGS (p<0.001, Figure 2A) and STKM2 (p<0.001, Figure 2B) cells as compared to control cells. Our quantitative real time PCR data confirmed that the reconstitution of TFF1 upregulated mRNA expression of p53 downstream target gene, *NOXA*, but not *p53*, in AGS (p<0.05, Figure 2C) and STKM2 (p<0.01, Figure 2D) cells. Consistent with these data, Western blot analysis showed an increase in protein expression of p53 and its target, NOXA, in TFF1-expressing AGS (Figure 2E) and STKM2 (Figure 2F) cells as compared to control cells.

**Figure 3:**
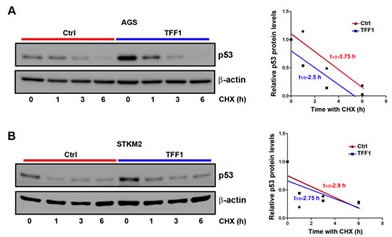
TFF1 expression does not promote p53 protein stability in gastric cancer cells AGS (A) and STKM2 (B) cells transiently expressing PTT5 empty vector or TFF1 were treated with cycloheximide (CHX) for the indicated time points, and subjected to Western blot analysis of p53 protein. The reconstitution of TFF1 does not decrease the rate of p53 protein degradation in AGS and STKM2 cells. These results are representative of at least three different experiments.

It has been well established that in response to stress stimuli, p53 protein is stabilized and activated by post-translational modifications [33]. To verify whether TFF1-induced p53 activation involves regulation of protein stability, we transiently transfected AGS and STKM2 cells with TFF1 or PTT5 empty vector for 48 hours, and subjected to Western blot analysis following treatment with cycloheximide (inhibitor of new protein synthesis) for different time points. The data indicated that the rate of p53 protein degradation was not significantly decreased by the reconstitution of TFF1 expression in AGS (Figure 3A) and STKM2 (Figure 3B) cell lines. These results clearly indicated that TFF1-induced activation of p53 does not involve regulation of p53 protein stability, prompting us to explore alternative mechanisms.

**Figure 4:**
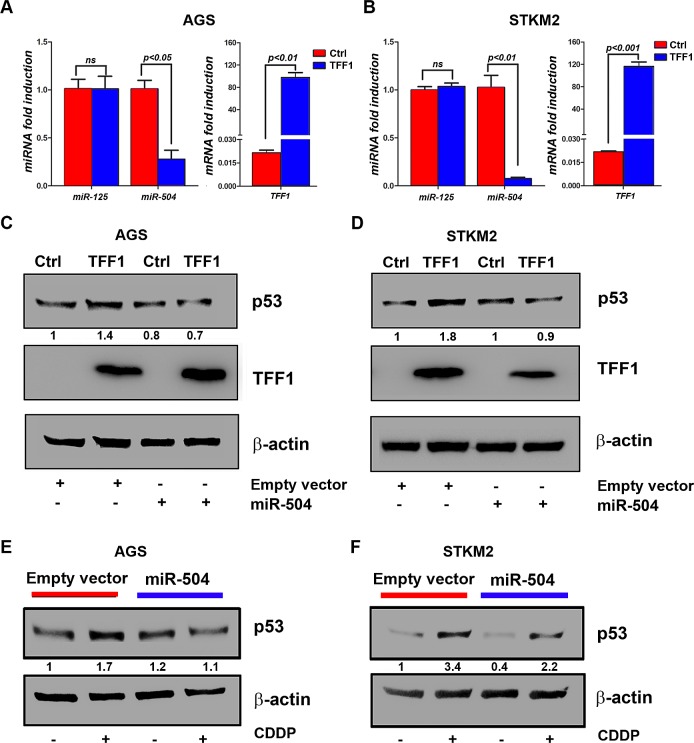
TFF1 activates p53 through down-regulation of miR504 expression AGS and STKM2 cells were transiently transfected with PTT5 empty vector or TFF1 and subjected to quantitative real-time PCR of mature miR-125, miR-504, and TFF1 48 hours post transfection. The miRNA levels of endogenous mature miR-125 and miR-504 were analyzed and normalized with miR-191. The reconstitution of TFF1 expression significantly reduced miR-504 expression, but not miR-125, in AGS (A) and STKM2 (B) cells; the levels of TFF1 mRNA expression are shown. AGS and STKM2 cells were transiently transfected with PTT5 empty vector or TFF1 in combination with control empty vector or miR-504 expression vector, and subjected to Western blot analysis. Overexpression of miR-504 reduced TFF1-induced p53 protein expression in AGS (C) and STKM2 (D) cells. AGS and STKM2 cells were transiently transfected with control empty vector or miR-504 plasmid for 48 h, treated with CDDP for 6 h, and subjected to Western blot analysis of p53. The reconstitution of miR-504 reduced CDDP-induced p53 protein levels in AGS (E) and STKM2 (F) cells. The intensity ratios of the indicated p53 protein level relative to β-actin were calculated using the ImageJ software. These results are representative of at least three different experiments.

Recent studies showed that some microRNAs, such as miR-504 and miR-125, may directly regulate p53 by reducing its protein levels [28, 34, 35]. Therefore, we hypothesized that TFF1 down-regulates miRNAs levels, thereby inducing p53 protein expression. To test this hypothesis, we first checked the microRNA expression of miR-125 and miR-504 in response to TFF1 expression. The quantitative real time PCR data showed that the reconstitution of TFF1 expression significantly decreased miR-504 levels, but not miR-125, in AGS (p<0.05, Figure 4A) and STKM2 (p<0.01, Figure 4B) cells, suggesting that TFF1 activates p53 through down-regulation of miR-504. To validate the role of miR-504 in the induction of p53 by TFF1, we transiently transfected AGS and STKM2 cells with TFF1 or PTT5 empty vector in combination with control empty vector or miR-504 expression vector, and subjected to Western blot analysis. Our data indicated that the reconstitution of miR-504 significantly decreased TFF1-induced p53 protein levels in AGS (Figure 4C) and STKM2 (Figure 4D) cells. To confirm the role of miR-504 in suppressing p53 induction in a different model, we transiently transfected AGS cells with control empty vector or miR-504 expression vector and treated with vehicle or CDDP (10 μM) for 6 hours. The Western blot analysis data showed that the reconstitution of miR-504 suppressed CDDP-induced p53 protein level in AGS (Figure 4E) and STKM2 (Figure 4F) cells. Together, our data indicated that TFF1 induces p53 activation through down-regulation of miR-504, a negative regulator of p53, in gastric cancer cells.

### TFF1 induces apoptosis in gastric cancer cells

**Figure 5:**
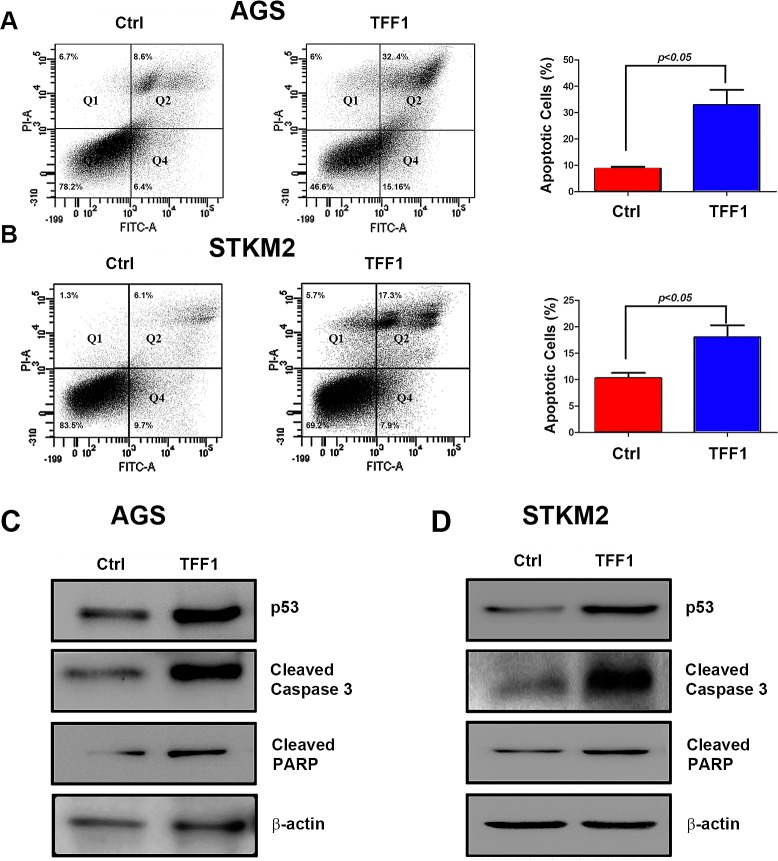
TFF1 induces apoptosis through activation of p53 AGS and STKM2 cells were transiently transfected with PTT5 empty vector or TFF1 and subjected to Annexin V and propidium iodide (PI) staining and FACS analysis. Representative FACS images of AGS (A, left panel) and STKM2 (B, left panel) cells and quantitative analysis of apoptotic cells (early apoptosis, Q4, + late apoptosis, Q2) are shown (A-B, right panels). Western blot analysis showing an increase in p53, cleaved caspase 3, and cleaved PARP protein levels in AGS (C) and STKM2 (D) expressing TFF1 as compared with control. The reconstitution of TFF1 induced significant levels of apoptosis in AGS and STKM2 cells. The data are representative of at least three different experiments.

In addition to inhibition of cell growth, we next examined the role of TFF1 in inducing apoptosis in gastric cancer cells. We transiently transfected AGS and STKM2 cells with TFF1 or PTT5 empty vector for 48 hours, and checked apoptosis by Annexin V and propidium iodide double staining followed by FACS analysis. Our results showed a significant increase of the combined early and late apoptotic cell populations in TFF1- expressing AGS (32.93%, p<0.05, Figure 5A) and STKM2 (18.05%, p<0.05, Figure 5B) cells as compared with control cells (8.73% and 10.28%, respectively). To elucidate the molecular basis of apoptosis we examined the pro-apoptosis mediators and molecular markers. Western blot analysis data revealed that the reconstitution of TFF1 significantly increased the protein levels of the cleaved form of PARP and cleaved caspase 3 in AGS (Figure 5C) and STKM2 (Figure 5D) as compared to control cells. These data clearly indicated that TFF1 induces apoptosis through activation of p53-caspases axis.

### TFF1 suppresses gastric cancer xenograft growth *in vivo*

**Figure 6:**
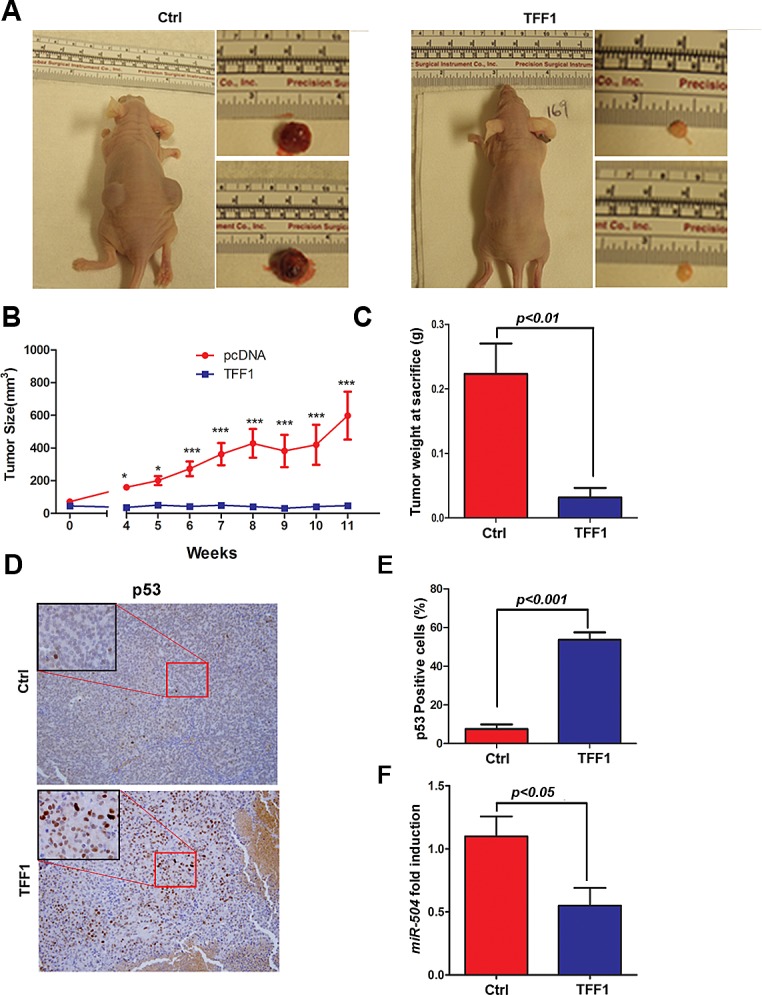
TFF1 suppresses tumor growth in a xenograft mouse model AGS cells stably expressing TFF1 or empty pcDNA vector (Ctrl) were subcutaneously xenografted into Nu/Nu nude mice. (A) Representative images of xenografted mice bearing tumors and dissected tumors are shown. (B) Tumor growth analysis indicated a significant increase of growth rate in control xenografts as compared to almost no growth in TFF1 xenografts. *p<0.05, ***p<0.001. (C) The quantitative analysis of weight of dissected tumors is shown. TFF1-expressing tumors were significantly smaller than control tumors (p<0.01). (D) Representative images of immunohistochemical staining of p53 in control and TFF1 xenograft tumors, original magnification (x200) and insets (x400) are shown. (E) The quantification of p53-positive cells expressed in percentage is shown. TFF1 xenografts displayed significantly more p53 staining than control xenografts (p<0.001). (F) Quantitative real time PCR indicating significantly less miR-504 expression in TFF1 xenografts than control xenografts (p<0.05).

To investigate whether TFF1 inhibits tumor growth *in vivo*, we used a xenografting mouse model. Briefly, we injected subcutaneously AGS cells stably expressing TFF1 or pcDNA empty vector into the flank regions of female athymic nude mice, and tumor growth was monitored. The control AGS cells generated large tumor masses in nude mice, whereas the TFF1-expressing cells either failed to generate tumors or generated significantly smaller tumor as compared to control (Figure 6A-B). Correspondingly, the average tumor weight In TFF1 group was decreased by 86% (p<0.01) relative to control nude mice group (Figure 6C). To confirm the antitumor effect of TFF1 *in vivo*, we performed immunohistochemical staining of p53 on tumor sections from TFF1 and control xenografts. Our data indicated a significant increase of p53 protein expression in TFF1 xenografts as compared to control xenografts (p<0.001, Figure 6D-E). Quantitative real time PCR data showed that TFF1 xenografts displayed a significantly less expression of miR-504 expression than control xenografts (p<0.05, Figure 6F). These results confirmed that TFF1 inhibits gastric tumor growth through activation of p53 and down-regulation of miR-504 *in vivo*.

## DISCUSSION

A large body of TFF1 literature has suggested *TFF1* as a tumor suppressor gene in gastric cancer [1, 3, 9, 29, 30]. In this regards, more than half of human gastric tumors lack TFF1 expression because of promoter hypermethylation, mutations or deletions of the *TFF1* gene [36, 37]. In a mouse model, loss of TFF1 promotes gastric dysplasia and adenocarcinoma [3, 8, 9]. Previous studies have shown that TFF1 inhibits growth and promotes apoptosis in gastric cancer cells [11, 12]. However, the mechanism by which TFF1 exerts these effects is still unclear. In this study, we investigated the tumor suppressor function of TFF1, and added additional clues concerning the role of TFF1 in gastric cancer. We have shown the effects of TFF1 on preventing tumor growth and promoting apoptosis by regulating the tumor suppressor gene *p53* through miR-504.

In our initial functional studies, we found that the reconstitution of TFF1 expression in gastric cancer cell models significantly decreased growth, indicating the tumor suppressor function of TFF1. Several reports indicated that activation of p53 by various stress stimuli leads to suppression of cell growth [32, 38, 39]. Therefore, we hypothesized that TFF1 function requires activation of p53 protein. Indeed, our results clearly showed that overexpression of TFF1 activated p53 as indicated by its increased protein expression and transcriptional activity. We then investigated how TFF1 activates p53 in gastric cancer cells. In response to stress, activation of p53 involves stabilization of the protein, which is mediated by extensive posttranslational modifications of p53 and protein-protein interactions with cooperating factors [22]. Hence, we checked whether TFF1 activates p53 through protein stabilization. Our data unequivocally demonstrated that TFF1-induced activation of p53 is mediated by a different mechanism. It has been reported that p53 can be transcriptionally upregulated in response to changes in the cell cycle caused by oncogenic activation [40-42]. Our data indicated that TFF1 overexpression has no significant effect on p53 mRNA levels, excluding transcriptional regulation as a molecular mechanism by which TFF1 activates p53 protein.

Protein levels and function of p53 have been shown to be regulated by miRNAs including miR-125 [43], miR-18 [23], and miR-504 [28]. The miR-504 has been reported as a negative regulator of p53 through its direct binding in the p53 3' untranslated region, thereby decreasing p53 function and protein expression, without affecting mRNA levels [28]. We postulated that TFF1 could activate p53 through down-regulation of miR-504 expression. In fact, our data demonstrated that the reconstitution of TFF1 expression significantly down-regulated endogenous miR-504, which led to increased p53 protein levels in gastric cancer cells. Furthermore, we confirmed that overexpression of exogenous miR-504 significantly abrogated TFF1-induced activation of p53, indicating that miR-504 mediates the effect of TFF1. However, since TFF1 is a secreted protein [44], the precise mechanism by which TFF1 regulates miR-504 expression to activate p53 requires further investigation.

In response to various cellular stresses, p53 induces apoptosis through primarily a transcription-dependent pathway that implicates expression of pro-apoptotic proteins (BAX, NOXA, and PUMA), and a transcription-independent pathway involving p53 translocation to mitochondria [45]. We investigated whether TFF1 activation of p53 is associated with induction of apoptosis in gastric cancer cells. Indeed, our data strongly suggest that TFF1 induces apoptosis through activation of p53, although we could not exclude TFF1 regulation of p53-independent pro-apoptotic pathways. Additional experiments will be required to investigate whether TFF1 could induce apoptosis in p53 deficient cells. We validated the *in vitro* data using a xenograft mouse model, showing that tumor growth was significantly suppressed in TFF1 xenografts as compared to control. Importantly, the growth suppression was associated with increased p53 protein expression and down-regulation of miR-504.

In summary, these findings highlight the role of TFF1 in suppressing growth and promoting apoptosis through activation of p53 in gastric cancer cells. We have demonstrated that the TFF1 activation of p53 involves down-regulation of miR-504 expression, a negative regulator of p53. This novel mechanism underscores the tumor suppressor function of TFF1 through activation of p53 in gastric epithelial cells.

## MATERIALS & METHODS

### Ethics statement

Experiments with mice were authorized by the Animal Care and Use Committee of the University of Vanderbilt and carried out according to the guidelines.

### Cell Culture and Reagents

Human AGS cells were obtained from American Type Culture Collection (Manassas, VA). STKM2 cells were a generous gift from Dr. Alexander Zaika, Vanderbilt University Medical Center. The AGS and STKM2, p53 wild type gastric adenocarcinoma cells, were maintained as a monolayer in Ham's F-12 culture medium (Gibco, Carlsbad, CA) supplemented with 10% fetal bovine serum (FBS) (Gibco). All cell lines were grown at 37°C in 5% carbon dioxide following the recommended procedures. Specific antibodies against p53 (DO1) was purchased from Calbiochem Millipore (Billerica, MA). NOXA, cleaved PARP, cleaved caspase3, and β-actin antibodies were obtained from Cell Signaling Technology (Danvers, MA). Human pre-microRNA Expression Construct Lenti-miR-504 from System Biosciences (Mountain View, CA).

### Reconstitution of TFF1 expression in cell lines

For stable expression, we established two cell lines, AGS and STKM2, stably expressing human TFF1. The human TFF1 coding sequence was amplified using PCR and cloned in-frame into pcDNA3.1 mammalian expression vector (Invitrogen Life Technologies, Carlsbad, CA) following standard protocols. AGS and STKM2 cells were transfected with pcDNA3.1-TFF1 or empty vector (control) using Fugene-6 (Roche Applied Science, Indianapolis, IN) following the manufacturer's protocols. Stably transfected cells were selected with G418 (0.5 mg/ml, Invitrogen).

For the transient expression of TFF1, AGS and STKM2 were transfected with the mammalian expression plasmid, pTT5 [46], in frame with TFF1, or PTT5 empty vector using Fugene-6 for 48 hours. The expression of TFF1 was analyzed by quantitative real time PCR and Western blot.

### Western blotting

Cell lysates were prepared in RIPA buffer containing Halt Protease and Phosphatase Inhibitors Cocktail (Pierce Biotechnology Inc.), and centrifuged at 4,390 g for 10 minutes at 4°C. Protein concentration was measured using a Bio-Rad Protein Assay (Bio-Rad Laboratories). Equal amounts of proteins (10–15 μg) from each sample were subjected to SDS/PAGE and transferred onto nitrocellulose membranes (Whatman, Boston, MA). Membranes were first probed with primary antibodies and subsequently with HRP-conjugated secondary antibodies. Protein bands detected by a commercial Immobilon Western Chemiluminescent HRP Substrate detection reagent (Millipore).

### Luciferase reporter assay

To monitor the transcriptional activity of the p53, we used PG13 Luciferase reporter containing 13 copies of the wild type p53-binding consensus sequence, and its mutant mPG13 (Addgene, Cambridge, MA). Fugene-6 was used for transfection as directed by the manufacturer's protocol (Roche Applied Science). AGS and STKM2 cells were transfected with PTT5-TFF1 mammalian expression vector or PTT5 empty vector in combination with PG13 and mPG13. As control for transfection efficiency, we transfected all cells with pSV-β-galactosidase plasmid. The transfected cells were incubated for 48 hours before assaying for luciferase activity and β-galactosidase activity as described previously [47]. The firefly luciferase activity was normalized to β-galactosidase activity and expressed as relative luciferase units with ± standard error of the mean (SEM).

### Colony formation assay

AGS and STKM2 cells stably expressing TFF1 or empty vector pcDNA were seeded at a density of 500 cells/well in six-well culture plates, and cultured at 37°C for 2 weeks. Cells were stained with 0.5% crystal violet solution, washed with PBS, and photographed. The images were analyzed using ImageJ (NIH) software.

### Xenografting in nude mice

To confirm TFF1 function *in vivo*, 2x10^6^ AGS cells stably expressing TFF1 or empty vector pcDNA were resuspended in a 200 μL DMEM-Matrigel mixture (50% DMEM supplemented with 10% FBS and 50% Matrigel), then injected subcutaneously into the flank regions of female athymic nude mice (Charles River, Wilmington, MA). After reaching 200 mm^3^, the tumor growth was monitored and measured twice a week and the tumor volume was calculated using the formula: Tvol=½ (L×W^2^) where Tvol is tumor volume, L is tumor length and W is tumor width [48]. All mice were sacrificed when the control group had tumors reaching a volume of 1000 mm^3^. The tumors were weighed, photographed, and subjected to immunohistochemistry, as described [8], using p53 antibody (Cell Signaling). All animal experiments were performed in accordance with institutional guidelines and were approved by the animal care review board at the University of Vanderbilt.

### Detection of cell apoptosis by flow cytometry

At 48 h after transfection, 5x10^5^ cells were harvested. According to the manufacturer's instructions for Annexin V-FITC kit (BD Bioscience, San Diego, CA), cells were resuspended in 500 μl of binding buffer and 5 μl of Annexin V-FITC followed by addition of 5 μl of propidium iodide (PI) and subsequent incubation for 10 min at room temperature in the dark. Apoptosis was evaluated by FACS analysis.

### RNA and miRNA extraction and quantitative real time RT-PCR

Total RNA was isolated using the RNeasy Mini kit (Qiagen, Germantown, MD), and single-stranded cDNA was synthesized using the RT-for-PCR Kit (Bio-Rad, Hercules, CA). Primers specific for human genes were designed using the online software Primer 3 (http://frodo.wi.mit.edu/primer3/). The forward and reverse primers were designed to span 2 different exons for each human gene (*TFF1*, *NOXA*, and p53). For miRNA extraction we used miRNA kit (Qiagen), and cDNA was synthesized following 3-step protocol: poly(A) tail synthesis, annealing of a poly(dT)-adaptor and reverse transcription [49]. Primers' sequences for qRT-PCR were obtained from the online database miRbase (http://www.mirbase.org/). All primers were purchased from Integrated DNA Technologies (Coralville, Iowa) (Table 1). The qRT-PCR was performed using an iCycler (Bio-Rad), with the threshold cycle number determined by use of iCycler software version 3.0. Reactions were performed in triplicate, and the threshold cycle numbers were averaged. The results of the genes and miRNA were normalized to the *HPRT* [50] and miR191 [49], respectively. Expression ratios were calculated according to the formula 2(Rt–Et)/2(Rn–En) [50], where Rt is the threshold cycle number for the reference gene observed in the test samples, Et is the threshold cycle number for the experimental gene observed in the test samples, Rn is the threshold cycle number for the reference gene observed in the reference samples, and En is the threshold cycle for the experimental gene observed in the reference samples. Rn and En values were calculated as an average of all reference samples.

**Table 1:** Human primers' sequences used in quantitative real time PCR

Gene	Ref. Seq. No.	Forward primer	Reverse primer
p53	NM_001126118	TAACAGTTCCTGCATGGGCGGC	AGGACAGGCACAAACACGCACC
NOXA	NM_001256067	AGAGCTGGAAGTCGAGTGT	GCACCTTCACATTCCTCTC
TFF1	NM_003225.2	GGTCCTGGTGTCCATGCTG	ACAGCAGCCCTTATTTGCAC

miRNA	Primer Sequence
Universal primer	GCGAGCACAGAATTAATACGAC
U6	GCAAGGATGACACGCAAAT
miR-191	CAACGGAATCCCAAAAGCAGCTG
miR-504	AGACCCTGGTCTGCACTCTATC
miR-125	TCCCTGAGACCCTAACTTGTGA

### Cycloheximide-based p53 protein stability assay

AGS and STKM2 cells were transiently transfected with TFF1 or PTT5 empty vector for 48 hours. Cells were treated with 100 μM of cycloheximide (Sigma, St. Louis, MO) and harvested at different time point (0, 1, 3 and 6 hours). Cells were lysed to generate whole cell lysates and subjected to Western blotting.

### Cell treatment with cisplatin (CDDP)

AGS and STKM2 cells were transiently transfected with TFF1 or PTT5 empty vector for 48 hours. Cells were treated with 10 μM of CDDP (Sigma) for 6 hours. Cells were collected, lysed and subjected to Western blot analysis.

### Statistical analysis

Using GraphPad Prism software, a 2-tailed Student's *t* test was used to compare the statistical difference between 2 groups. The results were expressed as the mean with ± SEM. The differences were considered statistically significant when the p value was < 0.05.
